# Morphological characterization and clinical effects of stromal alterations after intracorneal ring segment implantation in keratoconus

**DOI:** 10.1007/s00417-022-05572-2

**Published:** 2022-02-02

**Authors:** Loïc Hamon, Ursula Schlötzer-Schrehardt, Fidelis A. Flockerzi, Berthold Seitz, Loay Daas

**Affiliations:** 1 Department of Ophthalmology, Saarland University Medical Center (UKS), Kirrberger Straße 100, Bld. 22, 66421 Homburg, Saar Germany; 2 Department of Ophthalmology, Universitätsklinikum Erlangen, Friedrich-Alexander-Universität Erlangen-Nürnberg, Erlangen, Germany; 3 Department of Pathology, Saarland University Medical Center (UKS), Homburg, Saar Germany

**Keywords:** Keratoconus, Intracorneal ring segments, Ultrastructural changes, Lamellar channel deposits, Peri-segmental fibrosis

## Abstract

**Purpose:**

To analyze the histological and (ultra)structural stromal tissue changes after femtosecond (Fs) laser–assisted intracorneal ring segment (ICRS) implantation and their refractive and topographic effects in patients with keratoconus.

**Methods:**

This monocentric retrospective case series included 15 consecutive patients with clinical peri-segmental lamellar channel deposits after treatment with Fs-ICRS implantation for keratoconus. The stromal changes were investigated using in vivo confocal microscopy. Two patients underwent a penetrating keratoplasty after the Fs-ICRS implantation; the explanted corneas were processed for histopathology and transmission electron microscopy (TEM). Refractive and topographic effects were investigated comparing the uncorrected (UDVA) and corrected (CDVA) distance visual acuity, spherical equivalent (SE), flat (K1), steep (K2), and steepest (Kmax) keratometry before and after detection of lamellar channel deposits.

**Results:**

In vivo confocal microscopy revealed diffuse linear and focal granular hyperreflective structures. Histologically, there was mild proliferation of fibroblasts and fibrosis. TEM demonstrated focal accumulations of degenerated keratocytes with cytoplasmic lipid inclusions. There were no significant changes for UDVA (Δ = 0.0 ± 0.2 logMAR; *p* = 0.67), CDVA (Δ = 0.0 ± 0.1 logMAR; *p* = 0.32), SE (Δ 0.1 ± 0.9 D; *p* = 0.22), K1 (Δ = 0.3 ± 1.0 D; *p* = 0.28), K2 (Δ = 0.1 ± 0.9 D; *p* = 0.51), and Kmax (Δ = 0.3 ± 1.5 D; *p* = 0.17).

**Conclusions:**

Two types of structural stromal changes were identified: (1) diffuse peri-segmental fibrosis and (2) lamellar channel deposits. These structural changes showed no evidence of a relevant refractive or topographic effect.


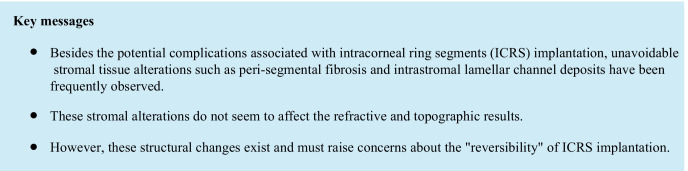


## Introduction

Intracorneal ring segments (ICRS) are crescent-shaped arcs of polymethylmethacrylate (PMMA) developed to be surgically inserted into the deep corneal stroma for the purpose of remodeling the corneal curvature. Originally developed to reversibly treat mild myopia [1–4], ICRS have been first introduced in 2000 by Colin et al. as an option to treat keratoconus (KC) patients [5]. Since 2004 and the approval of the Food and Drug Administration (FDA) for Intacs (Addition Technology Inc., Des Plaines, IL, USA) [6], the surgical procedure has been proven effective in improving the refractive and topographic outcomes of patients with KC [7–11], and the method has been extended to a larger spectrum of corneal ectasia, such as pellucid marginal degeneration (PMD) [12–14] and corneal ectasias after laser vision correction (LVC), e.g., after laser in situ keratomileusis (LASIK) [15, 16].

Currently, there are several ICRS available on the market, designed to be implanted into the 5–6 mm, 6–7 mm, or 7–8 mm optical zone of the cornea. The Intacs SK (for “Severe Keratoconus”) (Addition Technology Inc., Des Plaines, IL, USA) was developed for the 6–7 mm optical zone with the aim to correct larger myopic and astigmatic refractive errors in more advanced forms of corneal ectasia [17]. More recently, new designs with asymmetric progressive thickness emerged to treat KC with specific asymmetric phenotypes in corneal topography such as “duck” and “snowman” phenotypes [18, 19].

ICRS implantation is associated with potential complications such as infectious keratitis, asymmetric or superficial segment displacement, segment extrusion, posterior corneal perforations, corneal stromal edema around the incision, extension of the incision towards the central visual axis, halos, and glares [10, 20]. These complications have become rarer since the tunnel creation for the insertion of ICRS is no longer performed mechanically but commonly carried out using femtosecond laser (Fs laser) [20, 21]. Besides these complications, intrastromal structural changes such as peri-segmental fibrosis [22] and intrastromal lamellar channel deposits have been observed with slit lamp, in vivo confocal microscopy and optical coherence tomography [4, 23–27]. Still, the exact nature and mechanism of these structural changes remain currently unclear.

The purposes of this study were:

1. To characterize the morphological and (ultra)structural tissue changes of stromal alterations and deposits after Fs-ICRS implantation, including slit lamp examination, in vivo confocal microscopy, histopathology, and transmission electron microscopy (TEM).
2. To analyze the potential effect of stromal lamellar channel deposits on the refractive and topographic outcomes of the procedure.

## Materials and methods

This retrospective single-center study was conducted at the Department of Ophthalmology, Saarland University Medical Center in Homburg/Saar (UKS), Germany. The study was conducted in accordance with the Declaration of Helsinki and was approved by the local ethical committee (Ethikkommission der Ärztekammer des Saarlandes) with no. 202/20.

**Fig. 1:**
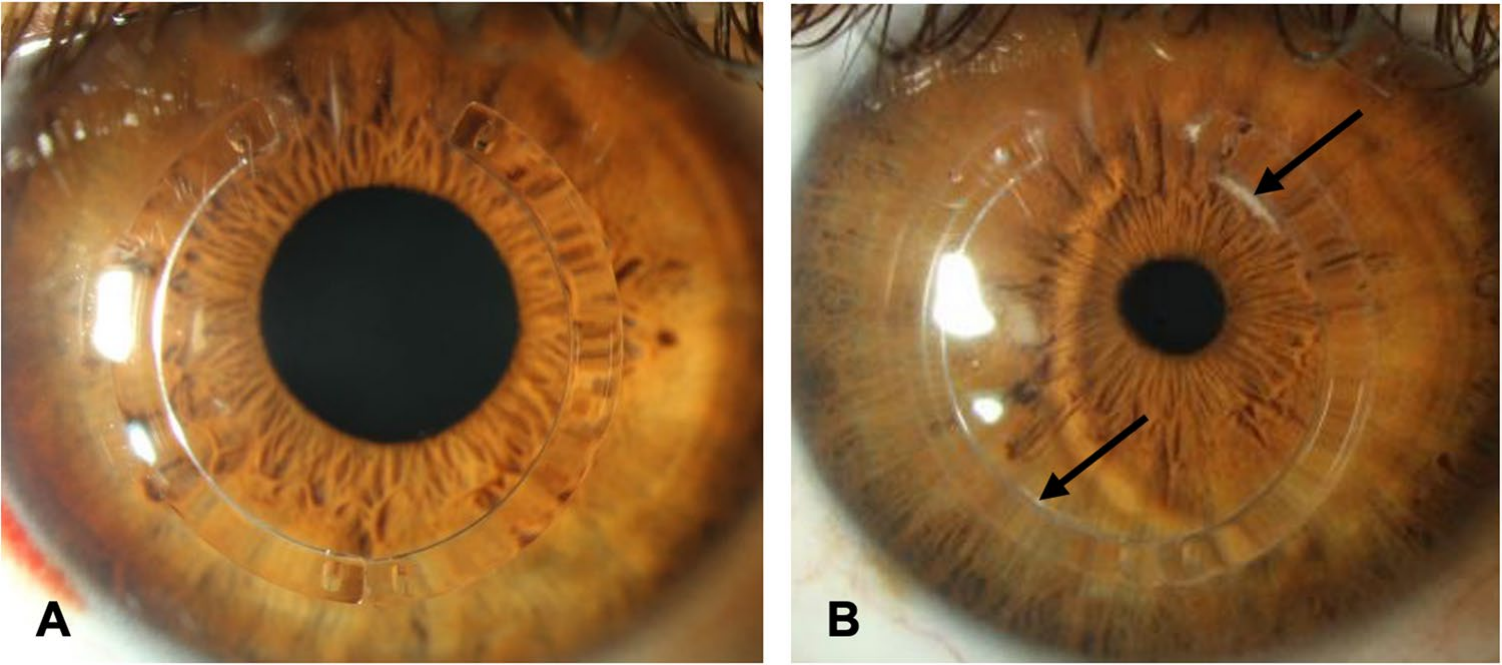
Slit lamp images of corneas after ICRS implantation to treat keratoconus. **A** Two intracorneal ring segments (ICRS) (Intacs SK) in the 6–7 mm optical zone, without manifest clinical evidence of intrastromal lamellar channel deposits. **B** The same two ICRS (Intacs SK) 3 years later with clinical manifestation of intrastromal lamellar channel deposits around the ICRS (arrows)

This study included 15 out of 160 patients (9.4%) from our Homburg Keratoconus Center (HKC) [28] treated with Fs-ICRS implantation for keratectasia between 03/2012 and 09/2020. The indication for Fs-ICRS implantation was an unsatisfactory CDVA combined with an intolerance to contact lenses. Before the implantation, each patient had a clear central cornea and a peripheral corneal thickness (PCT) of at least 450 µm in the 6–7 mm optical zone (implantation zone). All patients underwent a Fs laser–assisted ICRS implantation (Intacs SK—Addition Technology Inc., Des Plaines, IL, USA) in the deep corneal stroma (80% stromal depth) in the 6–7 mm optical zone between 03/2012 and 09/2020. The Fs laser technology (IntraLase FS laser; Johnson & Johnson Vison, Santa Ana, CA, USA) was used, with an energy of 1.5 mJ, to create the 360° circular tunnel in the deep stroma and the incision to insert the ring segments. Insertion was carried out manually. The following ICRS thicknesses and arc length had been individually selected, depending on the patient’s preoperative corneal astigmatism, coma, and ectasia topographic pattern, according to the manufacturer’s nomogram (AJL Ophthalmic S.A., Minao, Spain) [29]: 1 × 450 µm/150° (2 patients); 2 × 450 µm/150° (3 patients); 2 × 400 µm/150° (6 patients), 1 × 350 µm/150° + 1 × 210 µm/150° (2 patients); 1 × 400 µm/150° + 1 × 210 µm/150° (1 patient), and 1 × 450 µm/150° + 1 × 210 µm/150° (1 patient). Implantation of the ICRS was carried out in all 15 eyes without intraoperative complications at the Department of Ophthalmology at Saarland University Medical Center (UKS) in Homburg/Saar (Germany) by two experienced corneal/refractive surgeons [9, 10]. In all patients, a bandage contact lens (AIR OPTIX® Night&Day Aqua, Ciba Vision GmbH, Großwallstadt, Germany) was applied postoperatively for 1 week. Prednisolone acetate 10 mg/ml eyedrops (ED) and moxifloxacin hydrochloride 0.5% ED were applied alternately 6 times daily for 2 weeks. After 2 weeks, moxifloxacin ED were stopped and prednisolone acetate ED were then gradually reduced by 1 drop weekly. Preservative-free lubricant ED (Optive UD eye drops; Allergan Pharmaceuticals, Westport, Ireland) were additionally applied 6 times daily. All patients still presented a clear peri-segmental stroma at 191 ± 143 days postoperative and thereafter developed various degrees of intrastromal lamellar channel deposits, which were diagnosed with the slit lamp at 375 ± 217 days (Fig. 1A–B).

The uncorrected visual acuity (UDVA) [logMAR], (spectacle-)corrected distance visual acuity (CDVA) [logMAR], spherical equivalent (SE) [dioptries, D], flat (K1) [D] and steep (K2) [D] anterior keratometry (3.2 mm zone), and the steepest anterior keratometry (Kmax) [D] (measured using a Scheimpflug camera Pentacam HR (OCULUS GmbH, Wetzlar, Germany)) were compared for all 15 patients between before (absence of) and after (presence of) visible intrastromal lamellar channel deposits on slit lamp examination using a Wilcoxon signed-rank test. Statistical analysis was performed with SPSS Version 20.0.0 for Windows (SPSS Inc., Chicago, IL, USA). Values are expressed as mean ± SD (minimum–maximum). *P*-values < 0.05 were considered significant.

In vivo confocal imaging of the cornea was performed for 5 (33.3%) out of 15 patients, at the day of slit lamp diagnosis, using a confocal scanning laser ophthalmoscope Heidelberg Retina Tomograph HRT 3 with a Rostock Cornea Module for confocal cornea microscopy (Heidelberg Engineering GmbH, Heidelberg, Germany).

Two of these patients underwent a penetrating excimer laser–assisted keratoplasty [30, 31] (both 8.0/8.1 mm diameter, double cross stitch suture [32, 33]) 3.1 and 7.0 years after Fs-ICRS implantation due to high residual irregular astigmatism with decreasing visual acuity. The procedures were uncomplicated. We recovered the 2 explanted recipient corneal tissues with implanted ICRS segments for analysis.

The first explanted cornea was entirely fixed in neutral buffered 4% formaldehyde and processed for histopathological analysis. After embedding in paraffin, sections were obtained and stained with hematoxylin/eosin (H/E) and masson-trichrome staining [34].

The second explanted cornea was entirely fixed in 3% cacodylate-buffered glutaraldehyde and processed for TEM analysis. After post-fixation in 2% buffered osmium tetroxide for 1 h, the tissue was dehydrated and embedded in epoxy resin (Epon). Semi-thin and ultra-thin sections were cut on a Reichert-Ultracut (Cambridge Instruments, Nussloch, Germany), stained with toluidine blue, contrasted with uranyl acetate/lead citrate, and examined with an electron microscope (EM 906E; Carl Zeiss Microscopy, Oberkochen, Germany) [35].

## Results

The patient group included 11 males (73.3%) and 4 females (26.6%), mean age was 33 ± 12 years old. Thirteen (13) patients (86.6%) were treated for KC, 1 (6.6%) for PMD, and 1 (6.6%) for post-LASIK keratectasia. Postoperative values for analyzed parameters in the absence and presence of visible lamellar channel deposits on slit lamp examination are summarized in Table 1.

**Table 1:** Refractive and topographic outcomes before (in absence of [Abs.]) and after (in presence of [Pres.]) slit lamp detection of lamellar channel (LCD) deposits in patients treated with Fs-ICRS implantation

	UDVA [LogMAR]			CDVA [LogMAR]			SE [D]			K1 [D]			K2 [D]			Kmax [D]		
Patient	Abs. of LCD	Pres. of LCD	Δ^b^	Abs. of LCD	Pres. of LCD	Δ^b^	Abs. of LCD	Pres. of LCD	Δ^b^	Abs. of LCD	Pres. of LCD	Δ^b^	Abs. of LCD	Pres. of LCD	Δ^b^	Abs. of LCD	Pres. of LCD	Δ^b^
1	0.2	0.4	0.2	0.2	0.2	0	− 2.00	− 1.75	0.25	47.4	48.8	1.4	51.0	51.3	0.3	60.0	63.5	3.5
2	0.4	0.3	0.1	0.1	0.2	0.1	− 1.75	− 2.75	1.00	44.5	45.3	0.8	48.1	46.4	1.7	53.6	52.1	1.5
3	0.4	0.4	0	0.1	0.1	0	− 4.50	− 3.50	1.00	45.7	45.6	0.1	47.2	47.7	0.5	53.7	53.1	0.6
4	0.4	0.4	0	0.2	0.3	0.1	− 2.00	− 2.00	0	45.7	44.9	0.8	49.6	49.7	0.1	56.7	56.5	0.2
5	0.3	0.2	0.1	0.1	0.1	0	− 1.50	− 1.75	0.25	41.7	41.7	0	45.6	45.6	0	54.8	53.6	1.2
6	0.3	0.5	0.2	0.1	0.1	0	− 17.5	− 17.5	0	51.9	51.9	0	56.7	56.7	0	72.9	72.9	0
7	0.6	0.3	0.3	0.2	0.1	0.1	− 1.75	− 2.00	0.25	41.5	42.9	1.4	46.6	45.5	1.1	50.5	50.8	0.3
8	0.1	0.1	0	0.1	0	0.1	− 2.25	− 2.00	0.25	43.1	43.3	0.2	46.6	47.3	0.7	49.2	50.6	1.4
9	0.7	0.5	0.2	0.4	0.2	0.2	− 3.75	− 6.25	2.5	44.4	46.7	2.3	49.5	51.2	1.7	58.1	54.8	3.3
10	0.4	0.4	0	0.2	0.2	0	− 0.50	− 0.25	0.25	41.5	41.3	0.2	47.9	47.7	0.2	52.7	51.9	0.8
11	0.5	0.5	0	0.4	0.4	0	− 0.50	− 0.25	0.25	41.2	42.4	1.2	45.0	46.3	1.3	51.8	51.4	0.4
12	0.8	1.3	0.5	0.6	0.6	0	− 1.75	0	1.75	39.8	39.9	0.1	47.4	48.0	0.6	56.4	56.4	0
13	0.4	0.4	0	0.3	0.3	0	− 1.50	− 1.50	1.50	43.0	43.2	0.2	47.2	47.1	0.1	56.3	55.7	0.6
14	0.5	0.6	0.1	0.3	0.3	0	− 3.00	− 3.00	0	50.2	48.5	1.7	56.3	55.6	0.7	72.6	71.8	0.8
15	0.3	0.4	0.1	0.2	0.1	0.1	− 0.25	0.50	0.25	44.0	43.8	0.2	46.4	46.6	0.2	50.0	49.9	0.1
Mean SD	0.4 ± 0.2	0.4 ± 0.3	0.0 ± 0.2	0.2 **± **0.1	0.2 ± 0.2	0.0 ± 0.1	− 3.0 ± 4.2	− 2.9 ± 4.3	0.1 ± 0.9	44.4 ± 3.4	44.7 ± 3.2	0.3 ± 1.0	48.7 ± 3.5	48.8 ± 3.5	0.1 ± 0.9	56.6 ± 7.2	56.3 ± 7.3	0.3 ± 1.5
p^a^	0.67			0.32			0.22			0.27			0.51			0.17		

^a^Calculated with nonparametric Wilcoxon signed-rank test

^b^Absolute difference between the follow-ups with absence and presence of lamellar channel deposits

*Fs*, femtosecond (laser assisted); *ICRS*, intracorneal ring segment; *UDVA*, uncorrected distance visual acuity (logMAR); *CDVA*, (spectacle-)corrected distance visual acuity (logMAR); *SE*, spherical equivalent (D); *K1*, flat simulated keratometry (3.2 mm central) (D); *K2*, steep simulated keratometry (3.2 mm central) (D); *Kmax*, steepest simulated keratometry (overall) (D); *LCD*, Lamellar channel deposits; *Absence of LCD*, postoperative follow-up before slit lamp detection of lamellar channel deposits (191 ± 143 days postoperative); *Presence of LCD*, postoperative follow-up after slit lamp detection of lamellar channel deposits (375 ± 217 days postoperative); *SD*, standard deviation

**Fig. 2:**
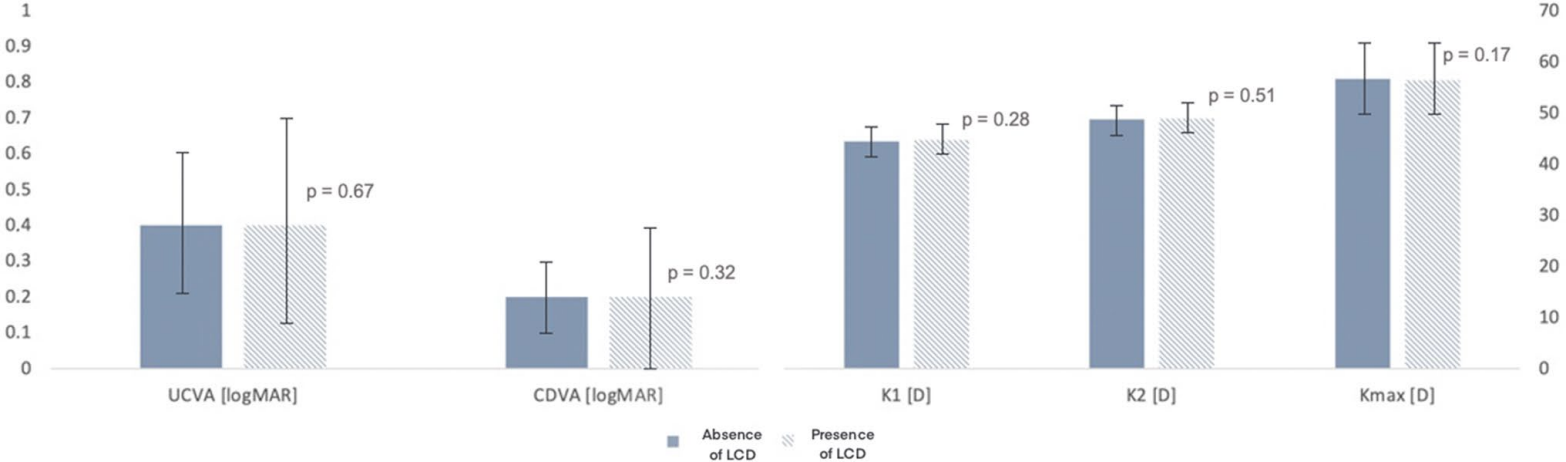
Comparison of UDVA, BCVA, and keratometry before and after slit lamp detection of lamellar channel deposits. UDVA, uncorrected distance visual acuity (logMAR); CDVA, (spectacle-)corrected distance visual acuity (logMAR); K1, flat simulated keratometry (3.2 mm central) (D); K2, steep simulated keratometry (3.2 mm central) (D); Kmax, steepest simulated keratometry (overall) (D); LCD, Lamellar channel deposits; Absence of LCD, postoperative follow-up before slit lamp detection of lamellar channel deposits (191 ± 143 days postoperative); Presence of LCD, postoperative follow-up after slit lamp detection of lamellar channel deposits (375 ± 217 days postoperative)

Comparing the postoperative values in the absence and presence of lamellar channel deposits (Fig. 2), neither the UDVA, with 0.4 ± 0.2 (0.1–0.8) logMAR in absence and 0.4 ± 0.3 (0.1–1.3) logMAR in presence of lamellar channel deposits (*p* = 0.67), nor the CDVA, with 0.2 ± 0.1 (0.1–0.6) logMAR in absence and 0.2 ± 0.2 (0–0.6) logMAR in presence of lamellar channel deposits (*p* = 0.32), showed significant increase or decrease after clinical manifestation of lamellar channel deposits. Mean SE also did not show significant changes with − 3.0 ± 4.2 (− 0.25 to − 17.50) D in absence and − 2.9 ± 4.3 (+ 0.50 to − 17.25) D in presence of lamellar channel deposits (*p* = 0.22). There were no statistically significant changes of both anterior surface main meridian K1 and K2, with a mean K1 of 44.4 ± 3.4 (39.8–50.2) D in absence and 44.7 ± 3.2 (39.9–51.9) D in presence of lamellar channel deposits (*p* = 0.28), and a mean K2 of 48.7 ± 3.5 (45.0–56.7) D in absence and 48.8 ± 3.5 (45.5–56.7) D in presence of lamellar channel deposits (*p* = 0.51). Mean Kmax also did not show significant changes, with 56.6 ± 7.2 (49.2–72.9) D in absence and 56.3 ± 7.3 (49.9–72.9) D in presence of lamellar channel deposits (*p* = 0.17).

**Fig. 3:**
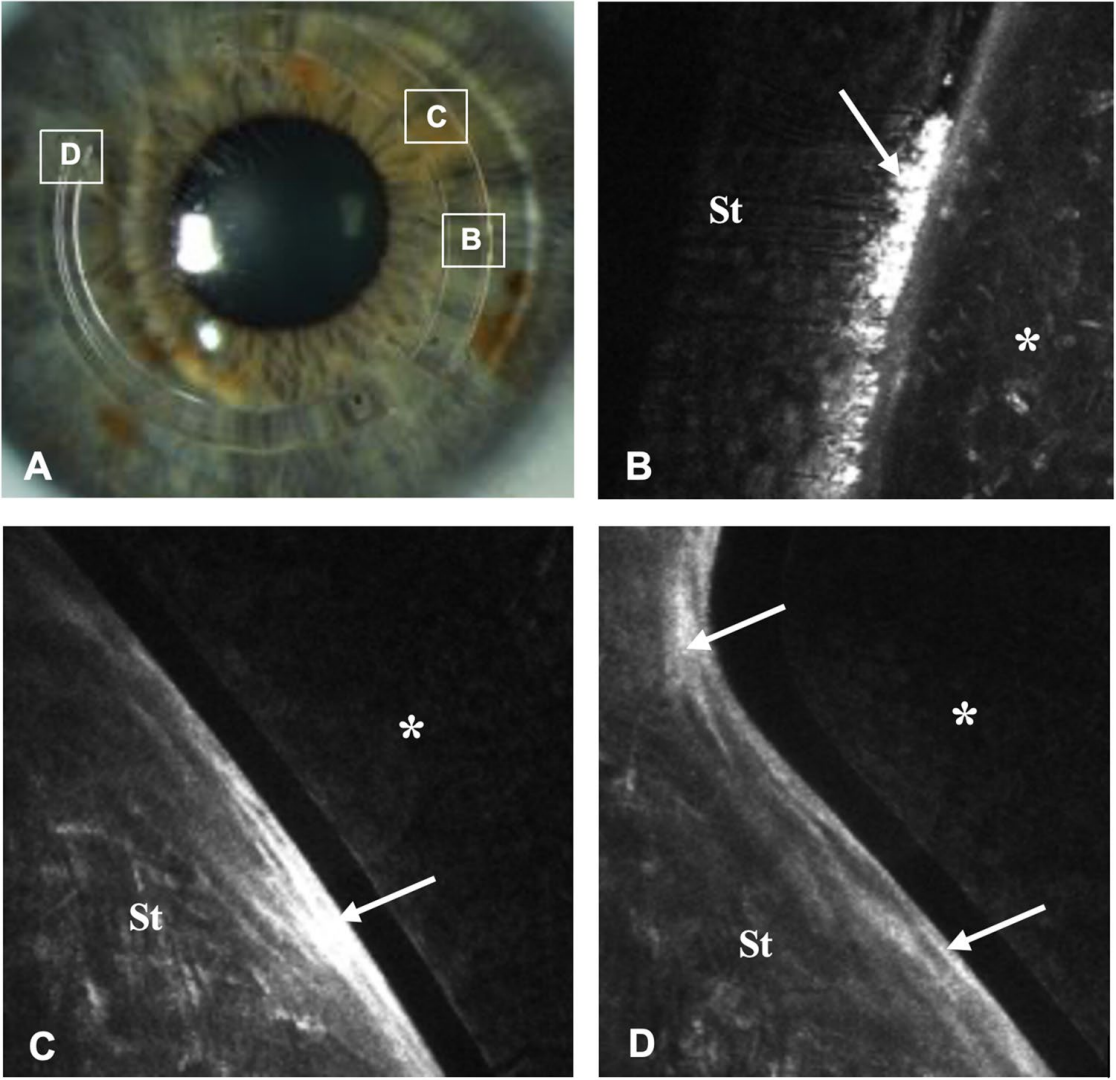
In vivo confocal microscopy of the stromal peri-segmental zone around ICRS. **A** Slit lamp image of the examined cornea with mild lamellar channel deposits (boxed areas show localization of in vivo confocal imaging). **B** Granular highly hyperreflective deposits of different sizes (arrow) in the free space between stroma (St) and ICRS (*), compatible with lipid inclusions. **C** Linear hyperreflective structures compatible with fibrosis (arrow). **D** Mild hyperreflectivity at the upper end of the segment, without typical granular or linear formation, compatible with mild fibrosis (arrows)

The in vivo confocal microscopy showed two types of hyperreflective structures (Fig. 3A–D): (1) focal granular highly hyperreflective structures following a peri-segmental lamellar formation, compatible with lamellar channel deposits; (2) diffuse linear mildly hyperreflective structures all around the ICRS, compatible with peri-segmental fibrosis.

**Fig. 4:**
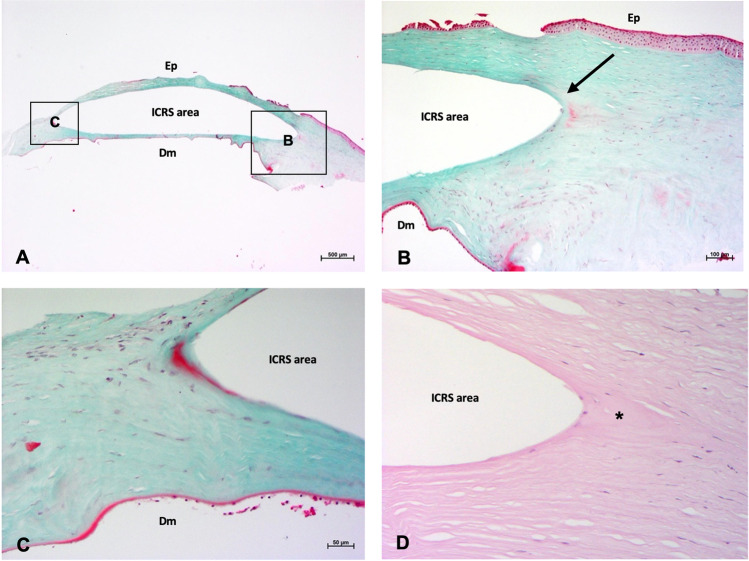
Histopathology of an explanted cornea with ICRS after penetrating keratoplasty. **A** Masson-trichrome staining: Overview (boxed areas show localization of higher magnification images). **B** Masson-trichrome staining: Mild proliferation of fibroblasts with mild fibrosis (arrow), no signs of inflammation or neovascularization. **C** Masson-trichrome staining: Moderate proliferation of fibroblasts without clear fibrosis, no relevant inflammation, no neovascularization. **D** H/E staining: mild fibrosis (condensed area *) (Ep, epithelium; Dm, Descemet membrane)

The histopathological examination of the first explanted cornea showed a proliferation of fibroblasts with mild fibrosis. As expected, no lipids could be visualized as these were dissolved during the preparation process (Fig. 4A–D).

**Fig. 5:**
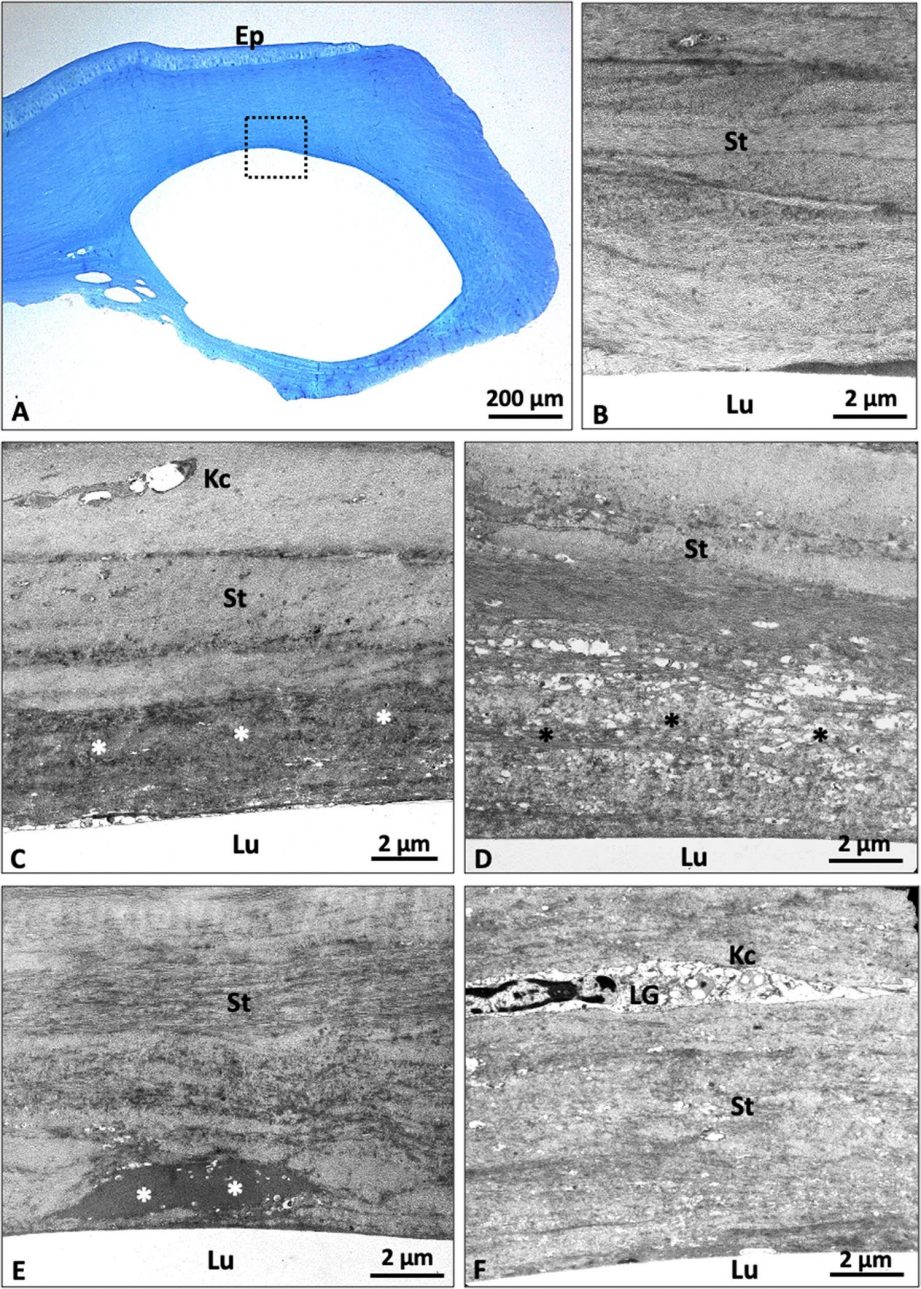
Transmission electron microscopy of an explanted cornea with ICRS after penetrating keratoplasty. **A** Overview in semi-thin section (boxed area shows localization of electron micrographs). **B** Normal collagenous stroma around the lumen. **C** Narrow zone (3–15 µm) of fibrotic stromal changes (*) with inclusion of amorphous material between collagen fibers and degenerative keratocytes. **D** Zone of fibrotic changes (*) with vacuolar inclusions. **E** Plaques of amorphous material (*). **F** Focal accumulation of cellular material, probably keratocytes. No inflammatory changes (Ep, epithelium; Kc, keratocyte; Lu, lumen; St, stroma)

**Fig. 6:**
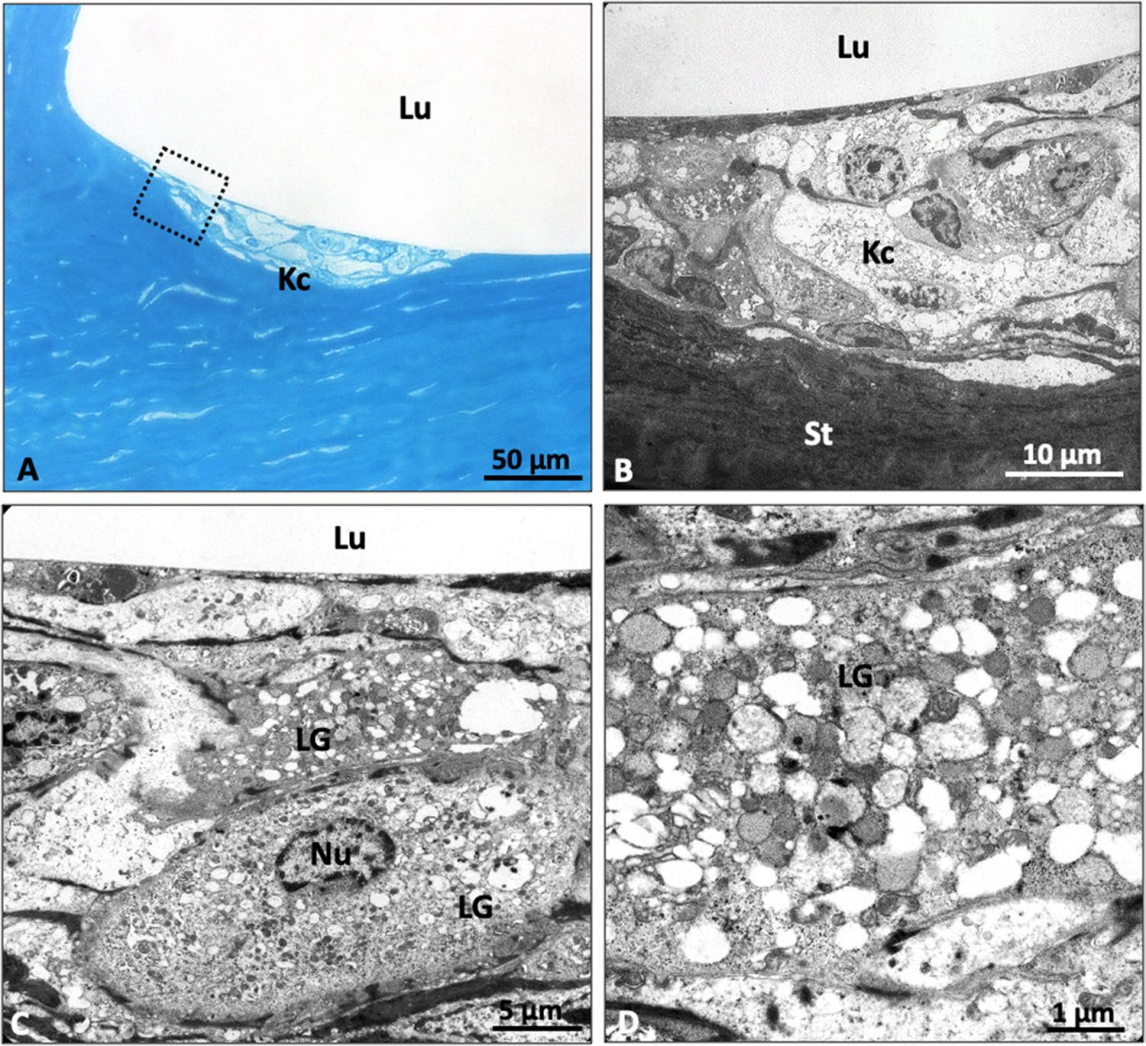
Transmission electron microscopy of an explanted cornea with ICRS after penetrating keratoplasty—zone with clinical evidence of linear channel deposits. **A** Overview in semi-thin section showing focal accumulation of foamy keratocytes in the border region to the ICRS (boxed area shows localization of electron micrographs). **B**, **C** Keratocytes showing accumulation of cytoplasmic lipid granules. **D** Intracellular lipid granules in detail (Kc, keratocyte; LG, lipid granules; Lu, lumen; Nu, nucleus; St, stroma)

TEM of the second explanted cornea showed peri-segmental fibrotic stromal changes within a narrow zone of 3 to 15 µm. Within this zone, amorphous and vacuolar materials were found to be interspersed between the condensed collagen fibers (Fig. 5A–E). Few degenerative keratocytes containing cytoplasmic lipid inclusions were occasionally observed in this zone (Fig. 5F). In regions of clinical lamellar channel deposits, focal accumulations of degenerative keratocytes containing large amounts of cytoplasmic lipid inclusions could be visualized (Fig. 6A–D).

## Discussion

Since 1991 and the first ICRS implanted in humans [36], significant improvements in design [25], tunnel creation [37], and segment insertion techniques have led to better post-surgical outcomes [10] and reduction of complications [21]. Nevertheless, unavoidable tissue alterations remain such as peri-segmental lamellar channel deposits or fibrosis.

These alterations were already described at the premises of the development of PMMA corneal implants in animal models, where crystalline deposits were observed [38, 39]. The hypothesis for those lamellar channel deposits was an abnormal cholesterol production induced by post-surgical stress on keratocytes. These deposits were also observed during the first human clinical studies [40]. Ruckhofer et al. analyzed these deposits using in vivo confocal imaging and scanning electron microscopy on explanted ICRS [24] and supported the hypothesis of lipid deposits in the free space between the ICRS and the stromal tissue. This hypothesis is reinforced as these deposits tend to disappear after removal of the segment [4]. Nevertheless, Ruckhofer et al. did not defend the hypothesis of lipid synthesis by stressed keratocytes but assumed a phagocytosis of lipids accumulated in the peri-segmental space by the keratocytes [25]. Twa et al. also analyzed those lamellar channel deposits in an explanted human cornea with history of ICRS implantation in KC, using histology (oil red O staining), TEM, and immunohistochemistry. They found evidence of saturated and unsaturated lipid droplets of cholesterol ester and triglycerides in the keratocytes adjacent to the ICRS, but no manifest extracellular accumulation [26].

The present study also reveals highly hyperreflective granular inclusions in the free space between the ICRS and the stroma in the in vivo confocal microscopy, compatible with lipid deposits, supporting the observations of Ruckhofer et al. in the early 2000s on eyes treated for mild myopia [23–25]. TEM analysis performed on the explanted cornea also showed intracellular lipid droplets, as well as extracellular vacuolar inclusions, which could represent—as demonstrated by Twa et al. [26, 39]—areas of previous cholesterol inclusions, extracted during the preparation of the tissue for TEM. Histological analysis did not reveal any lipid inclusion, probably due to the dissolution of these during the preparation process for light microscopy [41].

In addition to the lamellar channel deposits, we also pointed out the diffuse presence of condensed and linear fibrotic tissue around the ICRS in in vivo confocal microscopy, histopathology, and TEM. This fibrosis was already described after ICRS implantation but also after other types of refractive surgery such as myopic photorefractive keratectomy (PRK) [42] or laser in situ keratomileusis (LASIK) [43], or after corneal injury [44, 45]. It is supposedly a wound-healing reaction with remodeling of corneal stroma and collagen synthesis [46]. Samimi et al. characterized these fibers as type IV collagen [41].

Stromal fibrosis and lamellar channel deposits lead to structural changes in the stroma which could theoretically influence the postoperative refractive and topographic results. This issue has already been investigated by some authors who demonstrated a lack of significant impairment of outcomes [23, 25]. The present study showed no significant difference between postoperative examinations in absence or in presence of lamellar channel deposits for UDVA, CDVA, SE, K1, K2, and Kmax, remaining consistent with the existing literature.

Nevertheless, these structural changes exist and extreme cases of tissue alterations after ICRS implantation were reported [47]. This must raise concerns about the “reversibility” of ICRS implantation, a term historically used to promote the procedure as an option to treat mild myopia. ICRS implantation has already proven to be reversible regarding the refractive and topographic changes after ICRS explantation [48, 49]. Structural changes also appear to be partially reversible. Spontaneous reversibility of lamellar channel deposits has been observed, generally from 24 months postoperatively [50]. In the case of ICRS explantation, the lamellar channel deposits disappear [4] and the peri-segmental fibrotic tissue tends to normalize [41].

The present findings are consistent with the existing literature and suggest a comparable pathophysiology of stromal tissue alterations in corneas treated with (Fs-)ICRS implantation for mild myopia and for KC. The potential stiffening effects of these structural changes are still unclear and subject to controversy. Further studies, including biomechanical analysis, are necessary to evaluate the potential role of peri-segmental fibrosis on KC stabilization, even without additional riboflavin UV-A crosslinking.

## Data Availability

Data and material were provided and collected in the Department of Ophthalmology, Saarland University Medical Center (UKS), Homburg/Saar, Germany. Data will be made available after publication on reasonable request.

## Declarations

The authors attest that they meet the current ICMJE criteria for authorship.
